# In free-roaming dog populations, does egg-based oral rabies vaccination programmes result in improved bait efficacy?

**DOI:** 10.18849/ve.v8i4.644

**Published:** 2023-11-29

**Authors:** Cecilia Tat+

**Keywords:** BAITS, CANINE, DOGS, ORAL VACCINES, RABIES VACCINE, STRAY DOGS, TRANSBOUNDARY ANIMAL DISEASES, UNOWNED DOGS, VACCINE UPTAKE, VACCINE

## Abstract

**PICO question:**

In free-roaming dog populations, does an egg-based oral rabies vaccination (ORV) compared with meat and fish based ORVs result in improved bait efficacy?

**Clinical bottom line:**

**Category of research:**

Treatment.

**Number and type of study designs reviewed:**

Three non-blinded, control trials were critically reviewed.

**Strength of evidence:**

Weak.

**Outcomes reported:**

Variables assessed in this Knowledge Summary included the type of bait that stray dogs were most interested in, and whether or not the dog was successfully vaccinated by release of the vaccine sachet into the oral cavity.

**Conclusion:**

There is weak evidence to show that stray dogs prefer egg-based baits in compared to other bait types, despite egg-based baits allowing for more successful perforation of the vaccine sachet, and hence a higher chance of a successful oral rabies vaccination.

## How to apply this evidence in practice

The application of evidence into practice should take into account multiple factors, not limited to: individual clinical expertise, patient’s circumstances and owners’ values, country, location or clinic where you work, the individual case in front of you, the availability of therapies and resources.

Knowledge Summaries are a resource to help reinforce or inform decision making. They do not override the responsibility or judgement of the practitioner to do what is best for the animal in their care.

## The evidence

The outcome of this Knowledge Summary was to appraise bait efficacy, meaning which bait type a dog is most likely to uptake, and would allow for successful vaccination, by perforation of the sachet into the oral cavity.

Three non-blinded control trials were found, all of which had similar study designs, and supported the PICO question. The set-up of all the studies with large sample sizes and defined control groups allowed for solid evidence to be obtained. Gibson et al. (2019) only compared two types of bait constructs, whereas Chanachai et al. (2021) and Bonwitt et al. (2020) compared three different types of bait constructs providing greater comparison.

Gibson et al. (2019) evaluated two types of bait, egg and gravy-flavour, in stray dog populations in urban locations in India. Several variables were assessed such as type of bait, acceptance, consumption, sachet perforation, bait handling time, bait outcome and bait efficacy. Dog factors were also taken into account such as age, sex and size. This was similar to Bonwitt et al. (2020) who also evaluated dog characteristics, as these may affect preference towards the baits. Sample sizes were similar between Gibson et al. (2019) and Bonwitt et al. (2020) studies, allowing for more fair comparison. Chanachai et al. (2021) had a considerably larger sample size, and focused on other factors such as how the bait was offered and location of the dogs. However, there were discrepancies in how the bait was offered compared to the other two studies, as more baits could have been distributed on one day compared to another, meaning different numbers of dogs were offered any single bait type. All studies were similar in that they all noted down dog characteristics; how the bait was consumed or taken; whether the dog was interested in the bait, had swallowed the bait or had chewed the bait, and along with the time taken to chew the bait as well.

All studies assessed whether the bait was chewed, which allows for release of the vaccine into the oral cavity, an important factor to consider when assessing whether the vaccination was a success, especially in oral rabies vaccination (ORV). It should be noted that the Chanachai et al. (2021) study further assessed whether the sachet was also perforated, and Gibson et al. (2019) went even further by evidencing this perforation by the release of a food dye in the vaccine sachet, which coloured the oral cavity of the dogs; a visual factor that could be observed during the study and then noted down.

## Summary of the evidence

**Gibson et al. (2019):** 

Population:	Free-roaming dogs in locations chosen at random within two urban regions: Panjim and Goa Velha, India.
Sample size:	404 dogs.
Intervention details:	Two bait constructs included a placebo vaccine sachet incorporated in an egg-flavoured bait matrix or coated with a commercially available pet food gravy. The egg-baits were locally manufactured in India using ingredients and method as the proprietor bait from IDT Biologika, Dessau-Rosslau in Germany. Egg baits were then frozen in a foil zip-bag before use. Outer layer of the sachet used in the study is liquid absorbent and hence 15 sachets where placed in a zip-bag and before distribution, poured in and mixed with 100 g of commercially available chicken-flavoured pet food gravy, which coated the sachet. The capsule was made with biodegradable foil covered with a fleece that can absorb fluids. A colourant and sucrose dissolved in water was also added for detectability if contents of sachet had been perforated into the oral cavity for both baits. The field study took place on the 11 and 12 July 2018. Teams were trained for half a day on the bait handout method before being deployed. Three teams consisting of two people on moped distributed baits in randomly allocated sections of the study area between 7:00 and 17:00. Baits were defrosted shortly before each vaccination session. Type of bait offered to each dog was randomly pre-determined. Staff were trained to approach dogs indirectly, avoid eye contact, drop the bait in front of the animal from a distance and continue walking, then from a distance, record their observations.
Study design:	Non-blinded randomised control trial.
Outcome studied:	Bait interaction including type of bait, acceptance, consumption, sachet perforation, bait handling time, bait outcome and bait efficacy. Bait acceptance was defined as either sniffed, licked, ignored or did not acknowledge the bait. Consumption was recorded as whether the dog took the bait into the mouth, and what percentage of the bait was consumed (< 50%, > 50%, 100%). Time observed included duration of bait manipulation by the dogs (< 10, 10–30, 30–60 or > 60 seconds). Outcome of whether the sachet remnants were swallowed or discarded was also recorded. Bait efficacy was recorded as the staff’s assessment. The sachet contained active oral rabies vaccine (ORV), by observing the release of the dyed-water in the oral cavity. An unknown field was also included if the outcome was not observed (if the dog took the bait out of sight for example). Data was recorded whether the dog was alone, or with other dogs, and if so, with how many, as well as age, sex and size. Age was estimated by the staff (adult / juvenile / puppy).
Main Findings (relevant to PICO question)	Higher proportion of dogs consumed the egg bait than those offered gravy baits. 162/209 (77.5%) with egg baits. 134/195 (68.7%) with gravy baits. P value was 0.047, Chi^2^ value was 3.94, meaning results were significant. Where bait perforation status was observed, egg baits were perforated more often than gravy baits meaning greater chance of vaccine uptake. 133/162 (91.1%) with egg baits. 89/123 (72.4%) with gravy baits. P value was < 0.001, Chi^2^ value was 14.98, meaning results were significant.
Limitations:	Considerable variation between teams / staff which may show as an inconsistency in bait distribution methods. The consumption of the bait is highly influenced by the way the team approaches the dog and how they dropped / tossed the bait. Staff only had half a day of training and were not followed-up with supervision and evaluation to ensure competency and consistency in distribution of the bait. Monitoring methods was subjective and up to staff discretion, which may skew the results depending on the observer.

**Chanachai et al. (2021):** 

Population:	Free-roaming dogs in four municipalities, consisting of two municipalities in Rayong province (Choeng Noen, Phe), one in Phetchaburi province (Cha Um), and another in Nakhonsri Thammarat province (Thung Song). Another rural area in the eastern part of Thailand, Tapraya, was included on a later date.
Sample size:	1,930 dogs.
Intervention details:	SPBN GASGAS vaccine (Ceva Innovation Centre GmbH, Dessau,Germany) was used in these oral rabies vaccination (ORV). Stored at –20^o^C and transported using dry ice. Kept frozen in standard cool boxes at –18^o^C overnight before field use. Sachets were filled with the liquid vaccine virus. Two different bait types were used: manufactured egg-flavoured bait; locally produced intestine bait. The study team observed further optimisation of the egg bait by putting tuna or chicken-flavoured cat liquid snacks available from local markets on the outer surface immediately before presenting the bait to the dog. These baits were marked as egg+ baits. Intestine baits were prepared by inserting the sachet with the frozen vaccine into a segment of pork intestine. Placed immediately back in the freezer to prevent vaccine thawing. Sites for sampling was identified with the local municipality workers and dog caretakers to estimate free-roaming dogs could be found from each site. Vaccination team members received brief training on ORV, including vaccine bait handling, techniques for approaching dogs, methods for offering the vaccine bait, recording vaccine bait handling by individual dogs, interpreting effectiveness of vaccination attempt (defined as ‘perforated sachet or when dog chewed at least five times before swallowing bait and sachet’), and on retrieving the discarded vaccine sachet after bait consumption. Bait not accepted was recollected to prevent human contact with the baits. Oral vaccination campaigns were done in a week for each municipality. One bait type was allocated to a team in the study, and the team would decide whether to use the liquid snack on the egg bait depending on whether the dogs seemed to be difficult to access.
Study design:	Non-blinded control trial.
Outcome studied:	Group sizes of the dogs offered the ORV. How the bait was offered (directly to the dog, dropped in front of the dog when passing by, tossed / thrown to the dog). Dog demographic data (sex, age, size, single or together with other dogs). Type of staff offering the bait: municipality or Department of Livestock Development staff; dog caretakers; animal / public health volunteers. Percentage of dogs interested in the bait offered that chewed very shortly (< 10 seconds), swallowed the sachet, and considered vaccinated (defined as ‘perforated sachet or when dog chewed at least 5 times before swallowing bait and sachet’) per bait type. Location / site of the dogs. Vaccination coverage in study areas.
Main Findings (relevant to PICO question)	Most dogs were interested (meaning uptake of the vaccine bait) in the intestine bait, followed by the egg+ bait, with the egg bait garnering the least interest: 1209/1302 (92.9%) for intestine bait; 256/276 (92.8%) for egg+ bait; 288/330 (87.3%) for egg bait; results were significant with p value being 0.002 and Chi^2^ value being 10.10 (between intestine and egg baits); results were also significant with the egg+ bait, p value was 0.04, Chi^2^ value was 4.34). Intestine bait was swallowed more often than the egg and egg+ baits which did not allow for release of the vaccine into the oral cavity.
Limitations:	Did not have means to evidence or assess objectively whether the animal had been successfully vaccinated. Vaccination teams directly involved in this study received limited training and had limited experience in approaching free-roaming dogs. Unequal distribution of each bait type, more dogs were offered intestine based baits rather than the other two types which may skew the results. Refused baits were collected and offered to the next dog, which would have altered the look, smell and shape of the bait potentially skewing results.

**Bonwitt et al. (2020):** 

Population:	Free-roaming dogs in four urban and peri-urban districts of Bangladesh (in the Dhaka and Chittagong divisions).
Sample size:	356 dogs.
Intervention details:	As oral rabies vaccines (ORV) were not licensed in Bangladesh, placebo ORVs were used only. This study had three types of baits: Fishmeal baits consisting of a block of vegetable fatty acids and fishmeal. Intestine baits created from locally purchased cow intestine. Boiled for 5 minutes then cut into 8–10 cm segments. Egg baits were made from egg powder and gelatin as described from the study from Bender et al. (2017). As this ORV study was conducted during a national rabies vaccination campaign, the ORV evaluators conducted this study 2–3 days after involvement in a capture-vaccinate-release programme. The evaluator stood in the centroid of one of the four areas, and a simple random direction generator was used to complete a transect. The evaluators were then given 60 baits and instructed to hand out at least 30 of the baits. Six evaluators participated in total, all had multi-year experience interacting with free-roaming dogs through national vaccination campaigns. Each evaluator was randomly assigned a bait type and then reassigned a different bait when moving to the next evaluation zone. Bait were all thawed before distribution. Baits were only offered to dogs that could be approached within a 3 metre distance, so that the bait could be thrown to the dogs. Dogs in crowded or unsafe locations such as a busy street were not selected. Dogs that were showing signs of aggression were also not offered bait. A mobile phone application was used to record the ORV data.
Study design:	Non-blinded randomised control trial.
Outcome studied:	When offered a bait, the evaluator recorded bait contact (‘showed interest’ or ‘ignored’), bait consumption, proportion of bait consumed (‘little’, ‘mostly’, or ‘complete’), and bait consumption time (recorded as the time elapsed between the dog being offered a bait and consuming it or losing interest, measured in time intervals (< 30, 30–60, 61–120 or > 120 seconds)). Dog characteristics including age, body condition, and temperament were also recorded. Age was an estimation by the evaluator. Temperament of the dog was determined at the discretion of the evaluator as well. Other factors were assessed: temperament of the dog (either as ‘accessible’ [if report as friendly] or ‘inaccessible’ [if timid or aggressive]); effectiveness (‘high-uptake’ or ‘low-uptake’); characteristics of the inaccessible dogs who consume baits, bivariate and multivariable log-binomial regressions were conducted. Independent variables of interest: bait attractants; age; body condition; site type (urban or peri-urban). Binary dependent variable of interest was bait uptake. Low-uptake baits were excluded from the analysis because and they were considered ineligible for future ORV campaigns.
Main Findings (relevant to PICO question)	Of the three baits: fish baits were ignored by 122/142 (86%) of dogs; 45/75 (60%) consumed the egg bait; 124/139 (89%)consumed the intestinal bait; P value was < 0.05 meaning these results were significant. Among the consumed baits: 10/18 (56%) of fish baits were fully consumed; 38/45 (84%) of egg baits were fully consumed; 122/124 (98%) of intestine baits were fully consumed; P value was < 0.05 meaning these results were significant.
Limitations:	Perforation of the sachet for vaccine release into the oral cavity was not tested in this study which usually tests for the efficacy of the vaccine. A vaccine pack could have negatively modified bait odour and consistency and hence bait uptake might be overestimated.

## Appraisal, application and reflection

As oral rabies vaccination (ORV) played a big part in the elimination of wildlife rabies in Europe, there was a potential for ORVs to be applicable in the Asian countries where rabies is endemic in the stray dog population (Müller & Freuling, 2018). Although the concept of ORVs can be applicable onto stray dogs, the application greatly differs to wildlife vaccination, due to the topography and ecology of stray dogs compared to wildlife. Most stray dogs live in urban areas and in close contact with humans, an important factor to consider when placing these baits. There is also the need to evaluate safety and offensiveness of the bait, due to potential human contact with stray dogs, as well as cultural considerations (as some countries in parts of the world are averse to certain meat products depending on religion and culture).

All studies summarised in this Knowledge Summary evaluating stray dogs interest in baits, revealed that the majority of dogs were interested in intestinal baits, with egg baits ranking second (or first in Gibson et al. [2019]) But it must be noted that intestinal baits were more often swallowed rather than bitten and perforated, meaning their use in ORV is, therefore, less effective compared to egg baits.

The Gibson et al. (2019) study, evaluated two types of bait, with the egg bait being shown a greater interest than the gravy bait. A benefit of this study was the use of a placebo-coloured dye in the sachets, enabling observers to assess definitively whether the vaccination was a success, compared to other studies. On the other hand, there was a lack of training for the staff, as most were from capture-vaccinate-release programmes, who only had half a day of training to get accustomed to a new method of vaccination. This may have led to discrepancies in the results due to subjective identification of the dogs, and the percentage of bait consumed.

The Chanachai et al. (2021) evaluated three types of bait: egg bait, intestinal bait, and bait with tuna or chicken flavoured snack (egg+) on top. It was found that intestinal baits had the highest interest out of the three. Vaccination rates (a successful vaccination defined as sachet perforation, or the dog having chewed the bait five times before swallowing), were highest in egg+ baits, followed by egg, then intestinal baits. The study also noted that intestinal baits were swallowed more frequently, compared to the other two baits, despite the high vaccination rate. This could potentially result in a lowered vaccination success, as more baits could be swallowed and not chewed, compared to the other egg baits. However, it should be noted that less dogs were interested in egg baits alone, which could also result in lowered vaccination success. Egg+ baits provided almost the same interest as intestinal baits, but a higher vaccination success rate, as well as a lowered swallowing rate, proving it more successful between the other two baits compared in the study. Again, with Gibson et al. (2019), there was limited training with staff, with evaluation, identification, and percentage of bait consumed being subjective, which might lead to discrepancies to appear between results. The biggest limitation was a lack of objectivity in assessing whether the vaccine was released into the oral cavity.

The study from Bonwitt et al. (2020), evaluated three different types of baits; intestinal, egg and fishmeal, with intestinal baits showing the highest rates of consumption. Though intestinal baits were shown to be eaten more quickly, and hence assumed to be swallowed, or give less vaccination coverage in the oral cavity compared to egg baits. This rendered this particular bait not as successful a bait type as the egg bait. But it is to be noted that with lower interest rates in egg baits, this would also mean a decrease in vaccination success. Evaluators were trained for 2 days, which was the most between the three studies, and a different bait was used whenever the evaluator went to another area. This meant there would be a lower rate of encountering the same dogs again compared to the other areas, where locations could have been potentially revisited. The major limitation in this study was that no sachet or vaccination product was used in this study, which can affect the taste, texture and smell of the product, potentially producing differing results in terms of bait preference.

In summary, all studies showed that although intestinal baits produced the greatest interest and consumption, egg baits were best in being consumed by stray dogs, and also in release of the vaccine into the oral cavity as it was most likely to be chewed for longer and perforated. However, to answer the PICO question, egg baits were not as effective in gaining interest and consumption from stray dog populations compared to intestinal baits, but if they were consumed they were more likely to successfully vaccinate a stray dog.

Dogs which chew the bait, and perforate the vaccine sachet, are deemed to be ‘vaccinated’, however, this assumption should be challenged in further studies. Instead, immunological status should be clarified via serological tests, and by evaluating titres of these dogs post oral vaccination in a test setting. Though when putting mass ORV to practice, this should not be undertaken as it would be unsustainable to do so.

## Methodology

**Search strategy:** 

Databases searched and dates covered:	CAB Direct on the CABI interface (1995–2023) Web of Science on the Clarivate interface (2018–July 2023)
Search strategy:	CAB Direct: ((("stray dog" OR "stray dogs" OR "strays" OR "free roaming" OR canine* OR canid*) AND (“egg based” OR egg Or “egg bait” OR bait) AND (“oral rabies vaccin*” OR “oral vaccin*”) AND (rabies OR lyssavirus*))) Web of Science: ((((((((((((((((((((((((((((((((((TI=(stray dog)) OR TI=(stray dogs)) OR TI=(canine*)) OR TI=(free-roaming)) OR TI=(free roaming dog)) AND AB=(egg)) OR AB=(egg-based)) OR AB=(egg-based)) OR AB=(egg bait)) AND AB=(oral rabies vaccin*)) OR AB=(oral vaccin*)) AND AB=(rabies)) OR TS=(lyssavirus*)) AND AB=(uptake*)) OR AB=(preference)) OR AB=(eat)) OR AB=(consume)) NOT AB=(wildlife)) NOT AB=(bat*)) NOT AB=(owned)) NOT AB=(ownership)) NOT AB=(owner*)) NOT AB=(cat*)) NOT AB=(fox))) OR AB=(egg-based oral rabies vaccination)) OR AB=(bait acceptance)) OR ALL=(rabies control)) OR AB=(stray dog)) OR AB=(stray dogs)) OR TI=(free roaming )) OR TI=(free-roaming)) OR TI=(oral rabies vaccin*)) AND TI=(oral vaccin*)) NOT AB=(raccoon*)
Dates searches performed:	21 Apr 2023

**Exclusion / inclusion criteria:** 

Exclusion:	Articles not in English, literature that included dogs that were in any way owned and wildlife canidae, literature that did not cover rabies, published literature with no focus on bait preferences with oral rabies vaccination, literature that explored parenteral routes of administering the rabies vaccines, review, opinion or commentary papers.
Inclusion:	Published paper on stray dog populations that had at least egg-based oral vaccinations and had results on preference rates of the bait, bait can be flavoured or made from raw food ingredient, oral vaccination was used as the basis of the intervention, literature was accessible and could be read in English and relevant to the PICO.

**Search outcome:** 

Database	Number of results	Excluded – Literature that included dogs that were in any way owned and wildlife canidae	Excluded – Literature that did not cover rabies	Excluded – Published literature with no focus on bait preferences with oral rabies vaccination	Excluded – Review, opinion or commentary papers	Excluded – Literature that explored the parenteral routes of administering the rabies vaccines	Total relevant papers
CAB Abstracts	8	4	0	2	0	0	2
Web of Science	55	15	5	23	8	1	3
Total relevant papers when duplicates removed							3

## ORCiD

Cecilia Pin Pin Tat: https://orcid.org/0000-0001-9079-6935

## Conflict of interest

The author declares no conflicts of interest.

## Disclaimer

Knowledge Summaries are a peer-reviewed article type which aims to answer a clinical question based on the best available current evidence. It does not override the responsibility of the practitioner. Informed decisions should be made by considering such factors as individual clinical expertise and judgement along with patient’s circumstances and owners’ values. Knowledge Summaries are a resource to help inform and any opinions expressed within the Knowledge Summaries are the author’s own and do not necessarily reflect the view of the RCVS Knowledge. Authors are responsible for the accuracy of the content. While the Editor and Publisher believe that all content herein are in accord with current recommendations and practice at the time of publication, they accept no legal responsibility for any errors or omissions, and make no warranty, express or implied, with respect to material contained within. For further information please refer to our Terms of Use.
